# Surgical resection of pancreatic insulinoma during pregnancy: Case report and literature review

**DOI:** 10.1016/j.ijscr.2019.07.019

**Published:** 2019-07-22

**Authors:** Carlos Castanha de Albuquerque Neto, Natália da Silva Lira, Marcelo A.R.C. Albuquerque, Fernando Santa-Cruz, Laís de França M. Vasconcelos, Álvaro A.B. Ferraz, Adriano C. Costa

**Affiliations:** a General Surgery Unit, Hospital das Clínicas, Federal University of Pernambuco, Recife, PE, Brazil; b School of Medicine, Federal University of Pernambuco, Recife, PE, Brazil; c Department of Surgery, Federal University of Pernambuco, Recife, PE, Brazil; d Oncology Unit, Federal University of Pernambuco, Recife, PE, Brazil

**Keywords:** Insulinoma, Pregnancy, Hypoglycemia

## Abstract

**Introduction:**

Insulinomas are neuroendocrine tumors characterized by an excessive secretion of insulin, and its most common primary site is the pancreas.

**Presentation of the case:**

Female, 31 years old, who underwent surgical resection of a pancreatic insulinoma measuring 0.5 × 0.5 cm during the third trimester of pregnancy. The patient started to present characteristic symptoms (hypoglycemia, adrenergic and neuroglycopenic symptoms) 18 months before being referred to our center. It was decided to resect the lesion due to the intensity of the symptoms and failure of the clinical treatment. The postoperative course was uneventful, and the fetus presented good vitality, as evidenced by the obstetric ultrasound. The patient was discharged from the hospital on the 6th postoperative day. The neonate was born at term, 38 weeks, with appropriate weight for gestational age. Today, around 12 months after surgery, the patient and the infant are in good health conditions and have no complaints.

**Conclusion:**

The moment of the surgical intervention in our case reflects the importance of this report, since it reinforces the feasibility of this procedure during pregnancy.

## Highlights

• Insulinomas represent an unusual but usually curable cause of hypoglycemia.
• Surgery is still the only possibly curative treatment for pancreatic neuroendocrine tumors.
• There are a limited number of cases of insulinoma treated during pregnancy.
• In all reported cases, both the woman and the child experienced good results.

## 1 Introduction

Insulinomas are tumors originating from pancreatic beta cells, which are located in the pancreas in 99% of cases. They are characterized by the excessive and rapid production of insulin with a long half-life that is not regulated by glycemia [1]. This type of neuroendocrine tumor represents an unusual but usually curable cause of hypoglycemia and accounts for 2% of all gastrointestinal tract neoplasms, with an annual incidence of 1–2 per 100,000 inhabitants [2,3]. It is more commonly found in females (60% of cases), between the fourth and sixth decades of life, and with a mean age at diagnosis of 45 years [[4], [5], [6], [7]].

Clinical manifestations of pancreatic insulinomas were described for the first time by Whipple and Frantz, and consist of a triad composed of hypoglycemic fasting symptoms, plasma glucose <50 mg/dL, and relief of symptoms following intravenous glucose administration [8]. During the insulin peak produced by these tumors, patients may present adrenergic symptoms, especially sweating, tremors, hyperphasia and palpitations [9,10]. In addition, neuroglycopenic symptoms may also occur, including mental confusion, visual changes, convulsions, and changes in consciousness level [11].

Insulinomas are the most frequent functioning endocrine pancreatic tumors, which may present malignant behavior in 10% of the cases and be associated with Multiple Endocrine Neoplasia type 1 (MEN1) in another 4–6% [12]. The recurrence rate in these is significantly higher after treatment, reaching about 21% in 10–20 years, whereas this rate in insulinomas not associated with MEN1 varies from 5 to 7% in 20 years [13].

Herein we present a case of pancreatic insulinoma operated on during the gestational period, together with a review of the literature on the diagnosis and treatment of these tumors. This work has been reported in line with the SCARE criteria [27].

## 2 Presentation of case

A 30-year-old female patient with no comorbidities and no family history of diabetes sought medical attention due to hypoglycemic symptoms associated with palpitations, perioral paresthesia, nausea, vomiting, and severe sweating during prolonged fasting periods for 3 months. She also reported an episode of syncope. A hemoglucotest was performed, showing capillary glycaemia of 35 mg/dL. Faced with this hypoglycemia, the team chose to perform an intravenous glucose infusion and keep the patient under observation. Upon improvement in the general condition of the patient, the frequency of meals was increased and prednisone was prescribed. At the same time, laboratory tests were requested in order to investigate the case better, but which were not performed by the patient.

There was partial relief of symptoms with prednisone, but the patient abruptly discontinued the medication due to pregnancy about 12 months after initiating treatment. The symptoms returned with great intensity two weeks after prednisone cessation. Thus, the patient chose to return to the outpatient Endocrinology clinic due to the recurrence of the symptoms.

At the consultation the patient was 31 years old and in her 10th week of gestation, and performed laboratory tests which confirmed hypoglycemia, having plasma glucose of 20 mg/dL. In addition, she underwent a prolonged fasting test with interruption after four hours (due to the onset of hypoglycemia), with increased C-peptide and insulin levels, confirming endogenous hyperinsulinemia (Peptide C = 1.93 ng/ml, VR ≤ 0.2 ng/mL; Serum insulin = 80 microU/ml, VR ≤3–25, blood glucose = 46 mg/dL and insulin/blood glucose ratio = 1.7) and suggesting insulinoma.

The team chose to hospitalize the patient due to the marked hypoglycemia and the intensity of the symptoms to try to clinically control the condition. However, conservative measures to control hypoglycemia were not sufficient, and continuous glucose infusion was necessary to keep the patient euglycemic. Due to the difficult management of the patient, an opinion was requested from General Surgery.

**Fig. 1:**
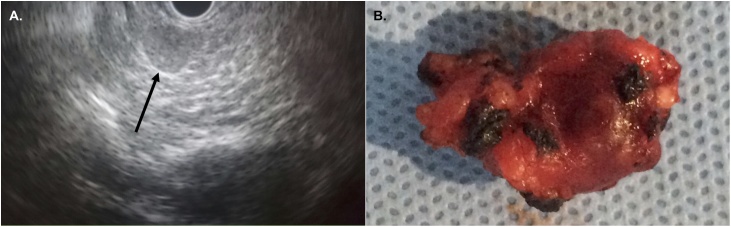
Endoscopic ultrasonography showing 11 mm lesion in the head-neck transection of the pancreas (arrow) **(A)**; surgical specimen: an irregular, brownish nodular tissue measuring 2.5 × 1.2 × 1.1 cm and weighing 2.0 g **(B)**.

The surgical team requested a diagnostic imaging evaluation through a total abdomen Ultrasonography (USG) and Nuclear Magnetic Resonance (NMR), which did not show pancreatic changes. An endoscopic USG was then performed, which was able to identify a single, well-defined, superficial and hypoechoic lesion measuring 11 mm, located at the head-neck transition of the pancreas (Fig. 1A).

Faced with the clinical treatment failure and intense discomfort on the part of the patient, the team opted for the surgical approach, even during gestation. The surgery was scheduled and performed in the 18th week of gestation, in March 2018. During surgery, an intraoperative USG located a single superficial lesion of the pancreatic parenchyma measuring 0.5 cm × 0.5 cm, unrelated to vascular structures, allowing enucleation of the tumor.

The patient developed hyperglycemia in the immediate postoperative period (glycemia = 213 mg/dl). A postoperative obstetric control USG was performed, which showed good fetal vitality. Thus, the patient was discharged on the 6th postoperative day in good clinical conditions, with cavitary drainage, in the presence of grade A pancreatic fistula, and without presenting new hypoglycemia episodes.

**Fig. 2:**
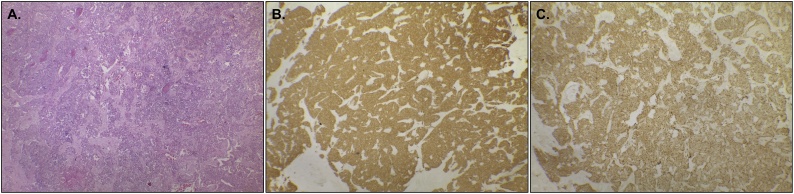
Neuroendocrine tumor of the pancreas stained with **(A)** Hematoxylin and Eosin; positive immunohistochemical staining for **(B)** synaptophysin and **(C)** chromogranin.

The surgical specimen was an irregular, brownish nodular tissue measuring 2.5 × 1.2 × 1.1 cm and weighing 2.0 g (Fig. 1B). The histopathological examination confirmed it to be a pancreatic neuroendocrine tumor, presenting 03 mitoses per 10 fields of great increase, and absence of angiopathic or perineural invasion, with free margins. The specimen’s immunohistochemistry was compatible with histological grade 2 neuroendocrine tumor, stained with hematoxylin-eosin (Fig. 2A) and showing a positive result for synaptophysin (Fig. 2B) and chromogranin (Fig. 2C) receptors, thus confirming insulinoma.

Childbirth occurred at term at 38 weeks, and the child was born healthy. At the moment, about 12 months after surgery, the patient remains euglycemic, with no signs of relapse and no other complaints.

## 3 Discussion

### 3.1 Laboratory evaluation

Biochemical diagnosis is performed by an inadequate elevation of insulin during a spontaneous or induced hypoglycemic episode [13]. Serum insulin levels higher than 5 IU/mL and serum glucose below 40 mg/dL with an insulin/glucose ratio equal to or greater than 0.3 reflect inappropriate insulin secretion in 98% of the cases [14,15]. In addition, serum C-peptide elevation is also useful in excluding causes related to the exogenous use of insulin because it evaluates the pancreatic reserve, so when it is increased it suggests endogenous insulin hypersecretion [7].

### 3.2 Diagnostic imaging

After the clinical diagnosis, the tumor should be located through the image to determine its staging and to plan the most appropriate therapeutic approach. In general, abdominal ultrasonography (USG) is performed as the initial examination. It is a simple, operator-dependent exam whose sensitivity varies between 23 and 63%, depending on the size and location of the tumor [6,9,16]. Computed tomography (CT) and Nuclear Magnetic Resonance (NMR) have higher sensitivity since the lesions are hyperdense in the contrasting phases. This is due to increased blood supply compared to other pancreatic tumors [9,16].

USG Endoscopy is an invasive and operator-dependent method. Its sensitivity for detecting pancreatic tumors which are not visualized in other methods such as abdominal USG and CT ranges from 82 to 93%. It has the advantage of not involving radiation in its performance, but is limited in penetration depth. The procedure also allows a concomitant biopsy with fine needle aspiration for histopathological study [10,14].

Somatostatin receptor scintigraphy, or OctreoScan, may aid in radiologic diagnosis. However, it may present many false negatives, since 50% of the insulinomas do not sufficiently express type 2 somatostatin receptors for their detection [13,16].

Positron Emission Computed Tomography (PET-CT) shows promising results. PET-CT with Gallium-radiolabeled peptides has presented better results than somatostatin receptor scintigraphy in some recent studies [5,10].

### 3.3 Treatment

Surgery is still the only possibly curative treatment for pancreatic neuroendocrine tumors. It is indicated to attenuate compressive symptoms of the tumor mass and systemic symptoms caused by the hormonal overproduction, as well as to avoid the malignant spread [17].

The surgical technique of choice is enucleation, which enables resecting insulinoma, preserving as much pancreatic tissue as possible [6,17]. Enucleation is indicated in benign, single, superficial, well-defined lesions which are smaller than 2 cm and are not related to the pancreatic duct. It is a safe technique with a low mortality rate; however, with morbidity similar to other pancreatic resections^17^,^18^].

When lesions are larger than 2 cm, poorly defined, locally advanced, suspected malignancy (lymph node spread or metastasis) or involving anatomical structures (vessels or biliary tract), it is prudent to opt for pancreatic resection techniques. Among these techniques are distal pancreatectomy, central pancreatectomy, and cephalic and total gastroduodenopancreatectomy [17].

Intraoperative USG can be used to increase surgical accuracy in lesions which have not been previously found or to confirm tumor location in case of doubt. In addition, it assists in choosing the best surgical technique, reducing the number of iatrogenic lesions [5,17]. This type of ultrasonography study has approximately 85% sensitivity and can also perform intraoperative staging [10,16].

An alternative for patients with high surgical risk is ethanol ablation. The procedure is guided by ultrasound and presents good resolution of hypoglycemia symptoms, but for an indefinite period [13]. In some cases, clinical treatment may be used in `both preoperative symptom control and in patients with surgical contraindication or patients with unresectable metastatic disease. Diazoxide, verapamil and phenytoin are used to minimize hypoglycaemic symptoms. Corticosteroids also help to stabilize glycemia at acceptable levels since they stimulate gluconeogenesis and insulin resistance [7].

Surgery should be avoided during pregnancy whenever possible due to increased risk for mother and fetus. Clinical treatment including dietary intake, diazoxide, calcium channel blockers and octreotide should be initiated to control the hypoglycaemia symptoms. The potential risks and benefits of treatment should be carefully considered due to the limited number of patients treated with octreotide during pregnancy. Intraoperative USG laparotomy should be performed in cases of failure of clinical treatment, especially in those cases in which the preoperative location failed [14].

### 3.4 Records in literature

There are 8 reports of patients surgically treated with pancreatic insulinoma during the gestational period in the literature. Surgery was performed in the first trimester in three reported cases, in the second trimester in another three, and at the time of cesarean section or soon after labor induction in two patients (Table 1). All women who underwent laparotomy during pregnancy, as well as their newborns, had good results. All these children were healthy and had normal development.

**Table 1:** Cases of surgically treated pancreatic insulinoma previously reported in the literature.

Cases	Author, year	Age (years)	Symptoms	Treatment
1	Serrano-Rios et al., 1976 [19]	37	Anxiety, sweating after overnight fasting, seizures, loss of consciousness.	Intravenous glucose, laparotomy in the 12^th^ week of gestation.
2	Rubens et al., 1977 [20]	21	Dizziness, loss of balance, diplopia, fatigue, mood changes, seizures, loss of consciousness.	Prednisolone, diazoxide and laparotomy in the 12^th^ week of gestation.
3	Wilson and Hugh, 1983 [21]	33	Perioral paresthesia and in the fingers after exercise, difficulty to wake up in the morning, nausea, vomiting.	Intravenous glucose and laparotomy in the 17^th^ week of pregnancy.
4	Friedman et al., 1988 [22]	37	Nausea, vomiting, altered consciousness, fulminant hepatic failure, hepatomegaly, ascites.	Intravenous glucose, laparotomy at the time of cesarean section (multiple hepatic implants).
5	Liberman et al., 1991 [23]	25	Loss of consciousness.	Laparotomy in the first trimester of pregnancy.
6	Atala and Tapia, 1992 [24]	24	Involuntary movements of the upper limbs, impulsive and aggressive behavior, loss of consciousness, seizures.	1st laparotomy in the 2nd trimester of gestation, and 2nd laparotomy after delivery (symptomatic persistence).
7	Auinger et al., 1994 [25]	26	Tremors, dizziness, weight gain (20 kg).	Intravenous glucose, laparotomy immediately after induced labor due to coma.
8	Bardet et al., 1994 [26]	25	Orofacial paraesthesia, 2 coma episodes.	Laparotomy in the 17^th^ week of gestation.

## 4 Conclusion

The surgical approach moment of the case described herein reflects the importance of this report, since it reinforces the viability of tumoral excision, even during the gestational period, without repercussions in the gestation or to the fetus during the intra and postoperative periods. The delivery in our case and in all previously reported in the literature occurred at term, with a healthy fetus and without sequelae.

## Funding

This research did not receive any specific grant from funding agencies in the public, commercial, or not-for-profit sectors.

## Ethical approval

Ethics approval was not necessary for this study and manuscript due to the type of study design (Case Report). All patient data and photographs are de-identified.

## Consent

Written informed consent was obtained from the patient for publication of this case report and accompanying images. A copy of the written consent is available for review by the Editor-in-Chief of this journal on request.

## Author’s contribution

Study concept and design: AABF, ACC.

Data Collection: CCAN, LFMV, MARCA.

Data Analysis and interpretation: CCAN, LFMV, NSL.

Writing the paper: FSC, MARCA, NSL.

Revision: FSC, AABF, ACC.

## Registration of research studies

Not needed.

## Guarantor

Adriano C Costa MD is the Guarantor of the study.

## Provenance and peer review

Not commissioned, externally peer-reviewed.

## Declaration of Competing Interest

The authors declare no potential financial conflict of interest related to this manuscript.
